# One-Step Partially Purified Lipases (*Sc*LipA and *Sc*LipB) from *Schizophyllum commune* UTARA1 Obtained via Solid State Fermentation and Their Applications

**DOI:** 10.3390/molecules22122106

**Published:** 2017-12-08

**Authors:** Yew Chee Kam, Kwan Kit Woo, Lisa Gaik Ai Ong

**Affiliations:** 1 Department of Biological Science, Faculty of Science, Universiti Tunku Abdul Rahman, Kampar 31900, Malaysia; kamyc@utar.edu.my; 2 Department of Chemical Engineering, Lee Kong Chian Faculty of Engineering and Science, Universiti Tunku Abdul Rahman, Kajang 43000, Malaysia; wookk@utar.edu.my

**Keywords:** sugarcane bagasse, used cooking oil, hydrolysis, esterification

## Abstract

Lipases with unique characteristics are of value in industrial applications, especially those targeting cost-effectiveness and less downstream processes. The aims of this research were to: (i) optimize the fermentation parameters via solid state fermentation (SSF); and (ii) study the performance in hydrolysis and esterification processes of the one-step partially purified *Schizophyllum commune* UTARA1 lipases. Lipase was produced by cultivating *S. commune* UTARA1 on sugarcane bagasse (SB) with used cooking oil (UCO) via SSF and its production was optimized using Design-Expert^®^ 7.0.0. Fractions 30% (*Sc*LipA) and 70% (*Sc*LipB) which contained high lipase activity were obtained by stepwise (NH_4_)_2_SO_4_ precipitation. Crude fish oil, coconut oil and butter were used to investigate the lipase hydrolysis capabilities by a free glycerol assay. Results showed that *Sc*LipA has affinities for long, medium and short chain triglycerides, as all the oils investigated were degraded, whereas *Sc*LipB has affinities for long chain triglycerides as it only degrades crude fish oil. During esterification, *Sc*LipA was able to synthesize trilaurin and triacetin. Conversely, *Sc*LipB was specific towards the formation of 2-mono-olein and triacetin. From the results obtained, it was determined that *Sc*LipA and *Sc*LipB are *sn*-2 regioselective lipases. Hence, the one-step partial purification strategy proved to be feasible for partial purification of *S. commune* UTARA1 lipases that has potential use in industrial applications.

## 1. Introduction

Filamentous fungi are suitable for solid state fermentation (SSF) due to their substrate colonizing mycelium, limited water tolerance and extracellular enzyme production. *Schizophyllum commune* is a commonly distributed split gill white-rot mushroom [1,2] found worldwide. It is cultivated in Malaysia, as it is popularly eaten by the Malay community [1]. However, it is also found to degrade wood and caused severe infections in humans [2]. Recently, Singh et al. [3] has purified the lipase produced by *S. commune* which was cultivated on *Leucaena leucocephala* seeds under solid state fermentation conditions. Due to these findings, *S. commune* was selected for this study.

The usage of lipase (triacylglycerol acylhydrolase EC 3.1.1.3) to convert water insoluble substrates is gaining more attention [4]. Enzymes are the alternative option in the continual pursuit for non-pollutant processes, moving in the direction of greener technologies [5]. As compared to chemical synthesis, biocatalysts or enzymes, which are classified as eco-friendly, are able to reduce the thermodynamic barrier that separates products from substrates and significantly lowers the energy consumption [5]. Studies of the selective triacylglycerol hydrolysis capabilities of lipases and their manipulations using diverse approaches have been reported [4] for various applications. Enzymes are a preferred choice due to their ability to work under mild conditions, ease of use, their production of less toxic by-products, and the fact the reactions are solvent-free or performed in an aqueous phase [5].

Lipases are suitable for working with raw, defined, sophisticated or unstable substrates under mild conditions and produce more stable products [5]. It is appealing that under certain conditions, lipases offer a natural approach compared to chemical catalysts [5]. Thus, this speed up the reaction processes and product procurement. The seafood industry generates wastes mainly from the discarded parts of fish, i.e., heads, fins, guts, scales and skins, that have potential for fish oil extraction and subsequent use in the food or nutraceutical industries [6] as an alternative to decrease land filling and environment pollution. Coconut oil, from the kernel of *Cocos nucifera* L., a clear liquid with pleasant aroma, is edible and used in bakery, confectionary, cooking, pharmaceutical and cosmetics [7]. On the other hand, butter has been in our daily lives since the early ages, and is a common item in the kitchen. In this study three raw triacylglycerols (TAGs), namely crude fish oil (long chain), coconut oil (medium chain) and butter (short chain), were selected for lipase hydrolysis studies.

TAGs are rich sources for the production of monoacylglycerols (MAGs) and diacylglycerols (DAGs). MAGs and DAGs are utilized as emulsifiers in cosmetics, drugs and food industry [8]. MAGs and DAGs can be chemically synthesized by glycerolysis of vegetable and animals lipids at high temperatures ranging from 200 to 290 °C, producing unwanted by-products [4,8]. Therefore, lipase is an alternative for lipids hydrolysis under mild conditions in the presence of excess water, generating lower wastes [8] and faster products production.

Besides hydrolysis, lipases also have the ability to esterify glycerol and free fatty acids to form TAGs [9]. As hydrolysis and esterification are reversible, water contents must be monitored in order to drive the reaction towards the desired products. Since lipases have specific affinity towards different substrates, thus acetic, lauric and oleic acids, which represent short, medium and long fatty acids, were chosen in this study for this purpose.

Thin layer chromatography (TLC) is a low cost reaction monitoring and an alternative method mainly used to detect chemical compounds, and it has been used for commercial characterization of oils for several decades [8,10,11], as it is rapid and sensitive. Junior et al. [8] recently studied the hydrolysis of triolein using Lipozyme RM IM from *Rhizomucor miehei* and the results were evaluated using TLC analysis.

According to Bayoumi et al. [12], high-purity of lipases is not required in the detergent industry, but rather the crude or partially purified version can be used as it is more cost effective. The industry is more concerned with the functionality and performance of the enzyme under certain conditions, rather than the enzyme purity. Ammonium sulfate was selected to precipitate and partial purify the crude lipase extract due to its inert effect of protein structure, solubility in water and being cheaply available [12]. Thus, the aim of this study is to evaluate the actions of one-step partial purified lipases on substrates of different chain lengths.

## 2. Results and Discussion

### 2.1. Optimization of Fermentation Parameters

In this study, eight variables i.e., inoculum density, moisture ratio, urea, incubation temperature, sugarcane bagasse (SB) solids, glucose, SB particle size and used cooking oil (UCO) ratio that affect lipase productivity were screened. The analysis of variance (ANOVA) was applied to test the interaction effects of the variables (Table 1). The *p*-value less than 0.05 indicated that the model terms are significant. Among the selected parameters, only moisture (*p*-value: 0.0021) and UCO (*p*-value: 0.0122) ratios were the significant variables. From these results, the correlations between the two factors and their effects on lipase production were evaluated using a 3-Level Factorial Design.

The model *F*-value of 14.24 (*p* < 0.0001) was obtained from the ANOVA analysis (Table 2), which implies the model is significant. There is only a 0.01% chance that such a “Model *F*-value” could occur due to noise. From the results, there is no correlation between the moisture and UCO ratios (*p*-value > 0.05).

**Figure 1:**
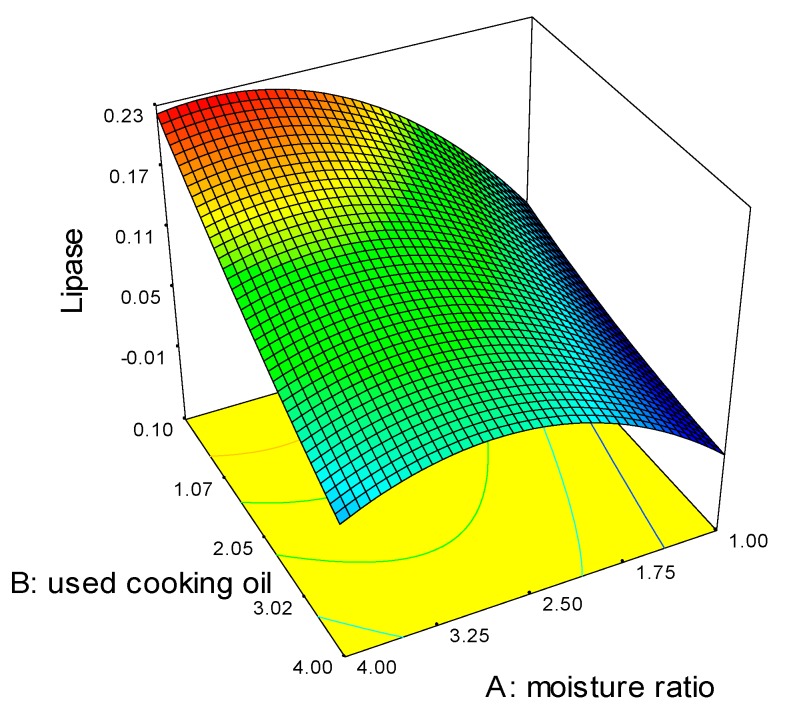
Response surface graph for the lipase production.

However, the moisture ratio was found to be the enhancing factor, while UCO ratio as the delimiting factor as shown in Equation (1) and Figure 1. The moisture ratio acts as the enhancing factor as all organism need moisture for growth. Moisture was needed only up to a certain extend in SSF, otherwise it will be submerged fermentation. On the other hand, the UCO ratio acts as the delimiting factor as too much oil will cause fungus suffocation, limiting the lipase production. UCO is only needed in small amounts, as an inducer.

Lipase activity = +0.047043 + 0.039508 Moisture ratio − 0.026561 UCO ratio(1)

By selecting a moisture ratio of 3.0, the predicted lipase activity is calculated as 0.112 based on Equation (1). After fermentation, the lipase activity was observed to be 0.110 ± 0.00 U/g_SB_. The predicted and observed values were found to be similar and the model was thus validated.

### 2.2. Partial Purification of Crude Enzyme Extract

**Figure 2:**
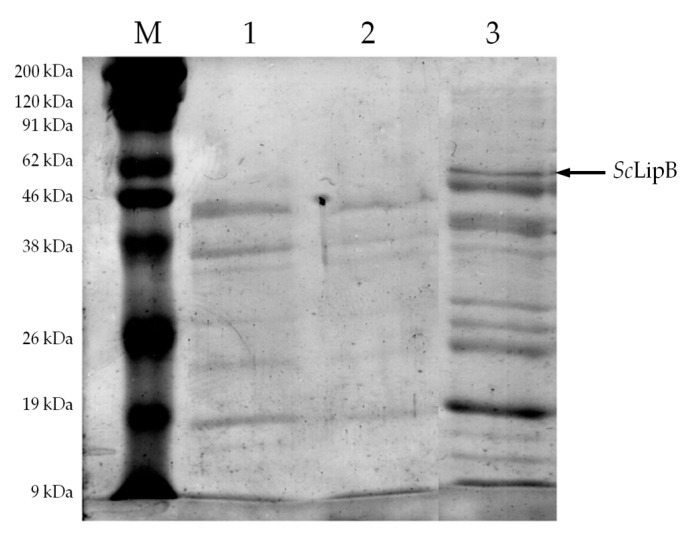
SDS-PAGE of *S. commune* UTARA1 lipase before and after partial purification. Lane M, Marker; Lane 1, Crude enzyme extract; Lanes 2 and 3, 30% and 70% (NH_4_)_2_SO_4_ precipitated fractions.

After ammonium sulfate precipitation and dialysis, it was observed that *S. commune* has two major protein fractions with high lipase specific activity (Table 3) which were obtained at 30% and 70% of the (NH_4_)_2_SO_4_ precipitation. Figure 2 shows the protein profile of *S. commune* lipase before and after partial purification. The 30% and 70% (NH_4_)_2_SO_4_ fractions were denominated as *Sc*LipA and *Sc*LipB, respectively. No activity was detected at 90% of (NH_4_)_2_SO_4_ precipitation and onwards, even though the protein contents were 3.68 mg and 73.88 mg, respectively. These results indicated that *S. commune* lipase did not precipitate after 80% of (NH_4_)_2_SO_4_ saturation. This is in agreement with the research done by Singh et al. [3]. They precipitated the lipase of *S. commune* ISTL04 up to 70% (NH_4_)_2_SO_4_ saturation, before further purifying it by Superdex^TM^ 200 gel permeation chromatography. They also found out that the protein size of *S. commune* lipase was 60 kDa, which coincides with Lane 3 in Figure 2. However, this protein size was not visible in Lane 2 of Figure 2. These results indicated that the partially purified fractions may contain different lipases. To further confirm this, each fraction was subjected to oil hydrolysis and glycerol esterification experiments to investigate their preference towards different substrates of various chain lengths.

### 2.3. Lipase Stability and Deactivation

**Figure 3:**
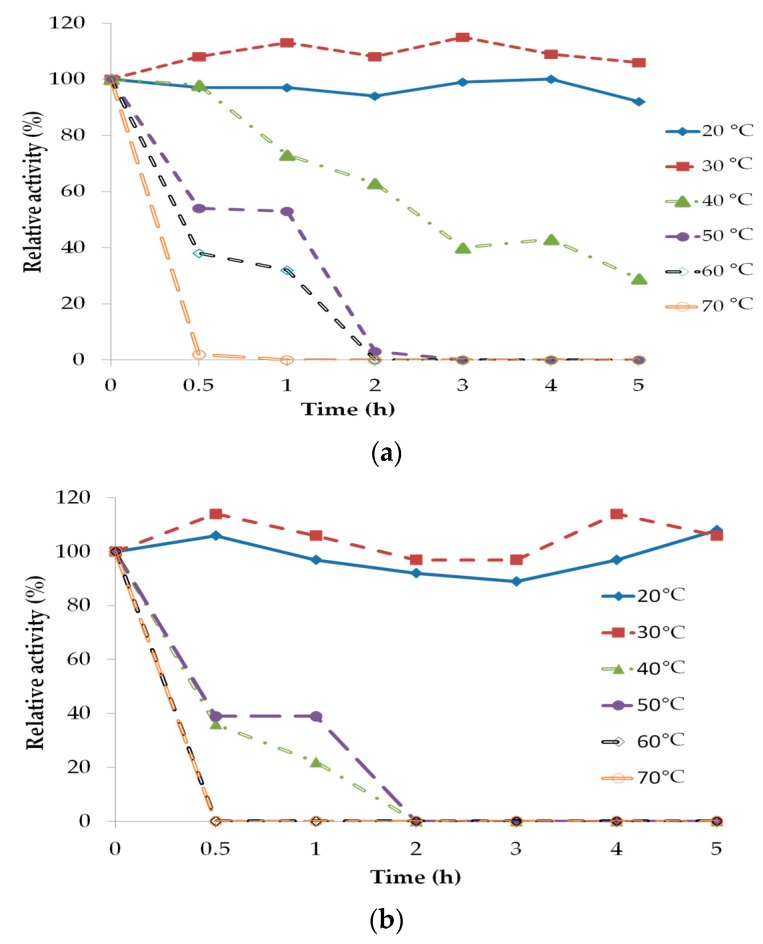
The stability and deactivation of (**a**) *Sc*LIpA and (**b**) *Sc*LipB after pre-incubation from 20 to 70 °C over a period of 5 h.

*Sc*LipA and *Sc*LipB were found to be stable at 20 and 30 °C over a period of 5 h (Figure 3). It was observed that after 30 °C pre-incubation, *Sc*LipA was activated, with over 100% relative activity. The relative activity of both *Sc*LipA and *Sc*LipB decreased as the incubation temperature and time increases. After incubated for 30 min at 60 and 70 °C, the relative activity has dramatically decreased to less than 50%. Thus, 30 °C was chosen for oil hydrolysis and glycerol esterification.

### 2.4. Oil Hydrolysis and Glycerol Esterification

In this study, undefined or raw triacylglycerol substrates which are often used in industry were chosen for hydrolysis. As TAG is associated with three fatty acids, all three fatty acids must be released before glycerol is detectable. From the results obtained, *Sc*LipA showed the highest affinity towards long chain triacylglycerol (LCTG) hydrolysis. This was apparent in Table 4 as the crude fish oil hydrolysis produced glycerol of 2.489 μmol/mg_protein_/day. Fish oil is a source of LCTG, especially long chain essential fatty acids (C20 and C22) [6]. The total crude fish oil from the extraction method was 13.2 g per 400 g of fish viscera and unwanted parts, which was used directly as crude fish oil. For this study, commercial coconut oil with 47.7% of lauric acid (C12:0) [13] was used as a model to represent the medium chain triacylglycerol (MCTG). Lauric acid was found as an effective antimicrobial and antifungal material [14]. As seen in Table 4, coconut oil hydrolysis produced glycerol of 0.405 μmol/mg_protein_/day. The result indicated that *Sc*LipA has an affinity towards MCTG as well. As for short chain triacylglycerol, unsalted commercial butter with significant amounts of butyric acid [14] was chosen for this study and the glycerol production was 0.236 μmol/mg_protein_/day. From the results, it was observed that *Sc*LipA showed affinity towards the hydrolysis of LCTG, MCTG and short chain triacylglycerol (SCTG).

*Sc*LipB catalyzed glycerol production from hydrolysis of crude fish oil (0.460 μmol/mg_protein_/day). However, glycerol production was not detected for the hydrolysis of commercial coconut oil and unsalted commercial butter. These indicated that *Sc*LipB has an affinity towards the hydrolysis of LCTG, but not MCTG and SCTG. These results also coincide with the findings of Singh et al. [15] on fatty acid methyl ester (FAME) production from cyanobacterial endolith *Leptolyngbya* ISTCY101 oil using immobilized purified 70% (NH_4_)_2_SO_4_ precipitated lipase from *S. commune* ISTL04. They reported that the cyanobacterial oil was mainly palmitic acid (C16:0), palmitoleic acid (C16:1), stearic acid (C18:0) and oleic acid (C18:1) fatty acids (more than 60%). The results from both studies verified that the 70% (NH_4_)_2_SO_4_ fraction from *S. commune*, which is similar to *Sc*LipB in this study, has affinity towards long chain fatty acids. This can also be observed as the *S. commune* UTARA1 was cultured on milled SB impregnated with UCO of palm oil origin which is rich in palmitic acid.

**Figure 4:**
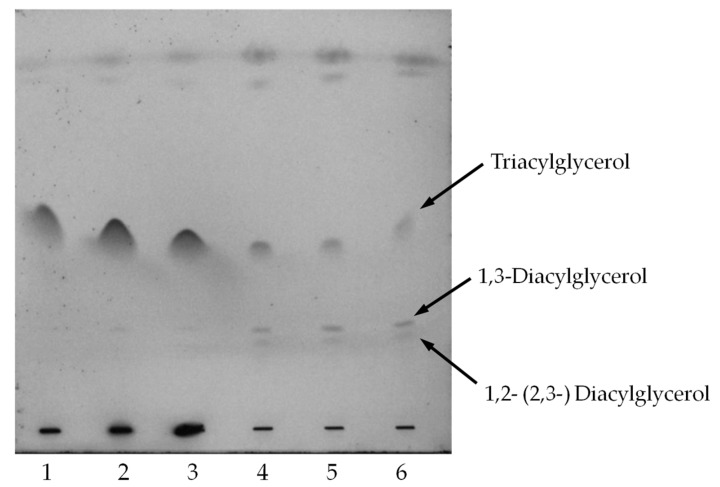
Time course profile of coconut oil hydrolysis by *Sc*LipA via TLC analysis. Lane 1, control; Lanes 2–6, Day 1–5.

Most lipases are either *sn*-1,3 regiospecific or non-regiospecific [16]. The *sn*-1,3 regiospecific lipase act on the ester bonds from the *sn*-1 or *sn*-3 positions of the TAGs [8], while non-regiospecific lipases hydrolyzes the ester bonds of the TAGs randomly [16]. On the other hand, Lotrakul and Dharmsthiti [17] stated that if the lipase is *sn*-2 regiospecific, 1,3-DAGs and 1(3)-MAG might be produced. According to Tong et al. [18], the TLC result obtained with *Candida antarctica* lipase A (CalA) displayed a clear 1,2- (2,3-) diolein spot and a slightly more intense spot of 1,3-diolein, indicating that CalA was a *sn*-2 regioselective lipase. For this study, similar scenario can also be observed on the TLC plate in Lanes 4–6 of Figure 4, showing a significant amount of 1,3-DAGs, and less of 1,2- (2,3-) DAGs. Therefore, it was stipulated that *Sc*LipA is most probably a *sn*-2 regioselective lipase.

**Figure 5:**
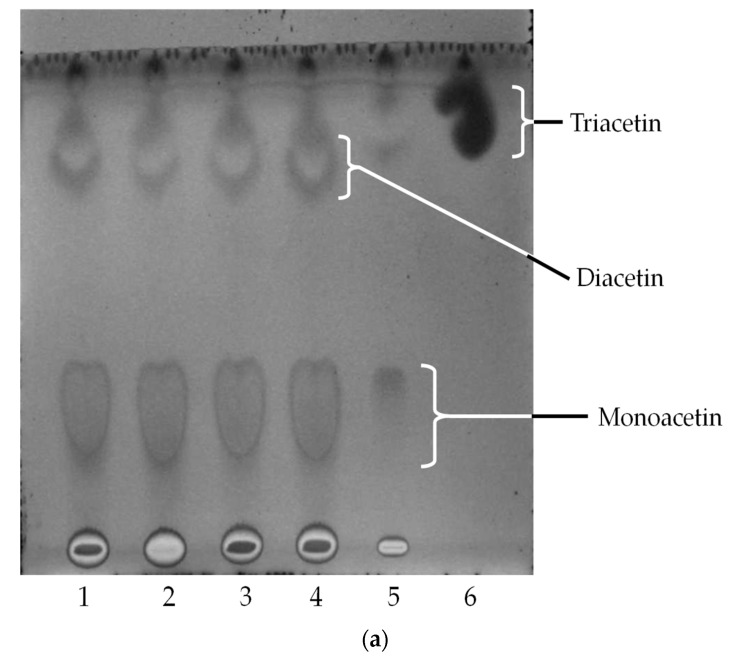

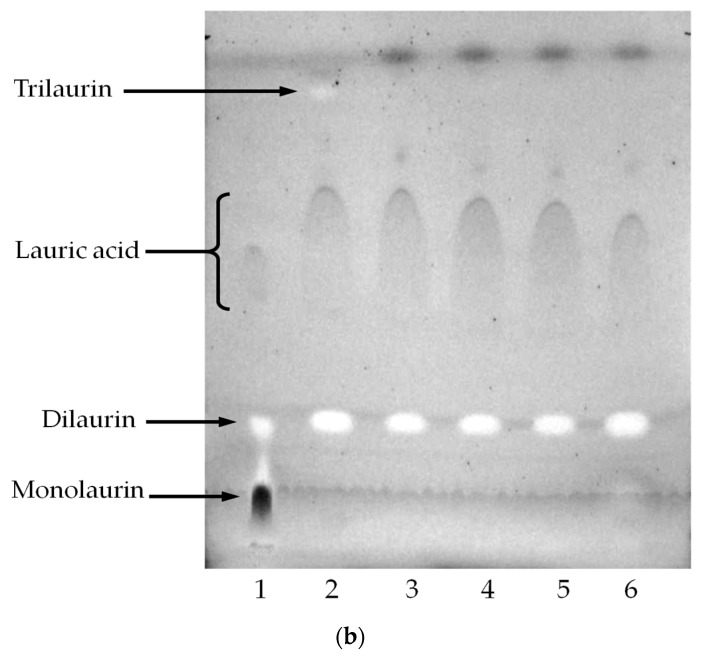
Time course profile of direct esterification of glycerol and (**a**) acetic acid; and (**b**) lauric acid with *Sc*LipA via TLC analysis. Lanes 1–5, Day 1–5; Lane 6, control.

TLC was recommended for qualitatively screening enzymatic TAGs hydrolysis reactions [8]. As a result of their affinity to a variety of substrates, lipases possess specific characteristics for producing different products [5]. Direct esterification of glycerol with acetic acid was carried out with *Sc*LipA in a solvent free system with the molar ratios of 1:9, and the results were evaluated using TLC. Figure 5a shows the time course profile of direct esterification of glycerol and acetic acid using *Sc*LipA and triacetin (Nacalai tesque, Kyoto, Japan) was used as a control. It can be seen that glycerol was esterified to form monoacetin and diacetin from Day 1–4; while triacetin was formed on Day 5 with a reduction in the intensity of monoacetin spot. As for lauric acid, monolaurin was synthesized on Day 1 as in Figure 5b Lane 1. Trilaurin can be observed on Day 2 for the molar ratios of 1:3 (glycerol:lauric acid); however, the trilaurin produced was degraded subsequently, as shown in Figure 5b Lanes 3–5. Hydrolysis and esterification are reversible in lipase reaction [9], where the water produced during esterification of lauric acid was most probably involved in the hydrolysis of trilaurin back to its original state, which is caused by the affinity of *Sc*LipA to hydrolyze MCTG. To ensure the reaction move towards the esterification process, water must be kept at a minimum amount or totally removed. This was also supported by Rosu et al. [9], who stated that water removal during reaction is very important for moving the reaction equilibrium towards esterification processes. Esterification of lauric acid and glycerol (molar ratio of lauric acid/glycerol of 3) with 9% Lipozyme IM 20 at 80 °C was conducted by Langone and Sant’ Anna, Jr. [19]. They found that 75% of trilaurin was produced after 26 h of incubation. Conversely, this study showed that trilaurin can be produced by Day 2 using 4% partial purified lipase at 30 °C; as the enzyme is more stable at this temperature. As for direct esterification of glycerol and oleic acid, *Sc*LipA did not show any triolein production (results not shown).

**Figure 6:**
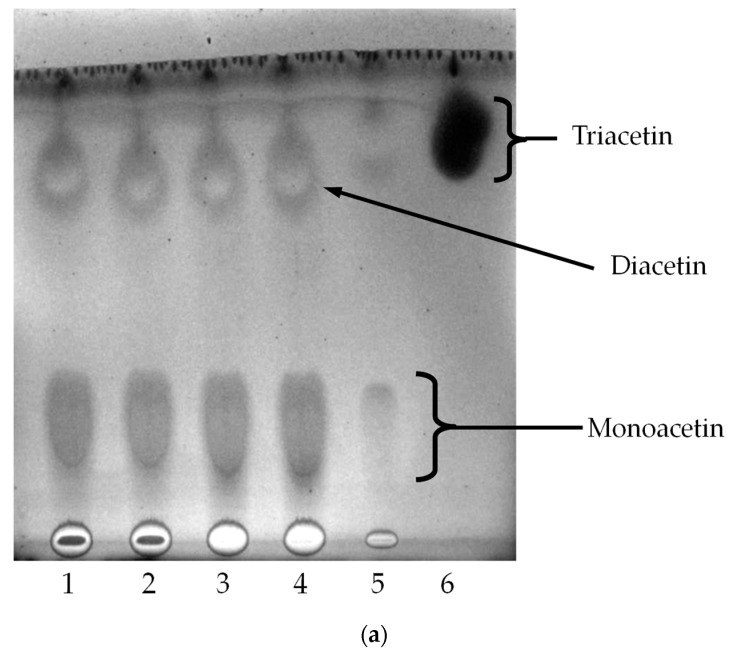

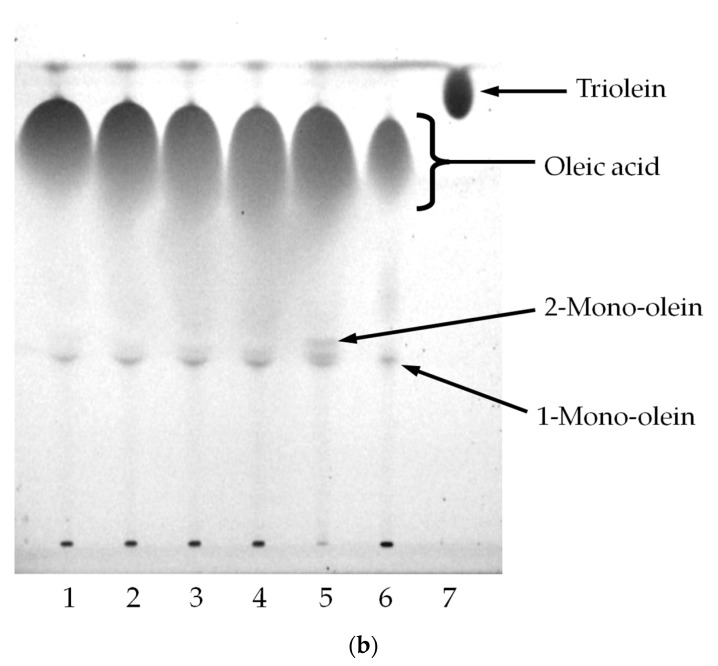
Time course profile of direct esterification of glycerol and (**a**) acetic acid; and (**b**) oleic acid with *Sc*LipB via TLC analysis. Lanes 1–5, Day 1–5; Lanes 6 and 7, control.

Figure 6a shows the time course profile of direct esterification of glycerol with acetic acid (molar ratios of 1:9) using *Sc*LipB. Triacetin (Nacalai Tesque, Kyoto, Japan) was used as a control. Similar with *Sc*LipA, *Sc*LipB also esterified glycerol forming monoacetin and diacetin from Day 1–4; while triacetin was formed only on Day 5, with a reduction in the intensity of monoacetin spot. Liao et al. [20] were able to obtain 100% triacetin conversion with the same molar ratio using Amberlyst A-35 at 105 °C for 4 h. They also stated that the glycerol conversion increased with the increase of temperature and molar ratio [20]. Conversely, this study took on a more subtle approach, using lipase at 30 °C.

In Figure 6b, oleic acid (Bendosen, Norway) and triolein (Sigma, St. Louis, MO, USA) were used as control for Lanes 6 and 7, respectively. With the molar ratio of 1:9 (glycerol:oleic acid), 2-mono-olein was only apparent on Day 5 by *Sc*LipB as shown in Figure 6b Lane 5, a distinct spot above 1-mono-olein. A *sn*-2 regioselective lipase will incorporate fatty acid at the *sn*-2 position in the glycerol molecule. Based on the results shown in Figure 6b, it was stipulated that *Sc*LipB is also a *sn*-2 regioselective lipase. Using *Aspergillus carneus* lipase at 30 s high power microwave irradiations (800 W, 90 °C) under solvent free system, higher percentage of triolein (50%) was synthesized [21]. Conversely, this study took on a more subtle approach, using lipase at 30 °C. By manipulating the reaction conditions and suitable enzyme selections, the resultant products can be controlled [8], driving the reactions either towards hydrolysis or esterification. As for direct esterification of glycerol and lauric acid, *Sc*LipB did not show any trilaurin production (results not shown).

Sharma and Rathore [22] stated that for bacteria lipases, ammonium sulfate precipitation can only purify the enzyme to a certain degree that is suitable for detergent formulations, but further purification is required for synthetic reactions. This might due to the fact that most bacterial lipases are intracellular, compared to the extracellular fungal lipases which may function well outside the cell. Based on our findings, the fungal lipases that were precipitated with ammonium sulfate can be used for several application purposes, namely TAGs hydrolysis and synthesis. To the authors’ best knowledge, this is the first time that *S. commune* was reported to have two different lipases, namely *Sc*LipA and *Sc*LipB. The two partial lipases were tested on various TAGs and fatty acids of different chain lengths and showed different affinities towards different substrates.

## 3. Materials and Methods

### 3.1. Fungal Cultivation and Solid State Fermentation

Isolate *S. commune* UTARA1 was maintained on potato dextrose agar incorporated with 1% of UCO at 30 °C. Four grams of milled SB were impregnated with UCO into a 250 mL flask before sterilization. Fermentation medium [23] was autoclaved separately before being added into each flask, maintaining the substrate to moisture ratio (g:mL). The flasks were then mixed before inoculation with seven mycelia discs prior to incubation at 30 °C for 5 days.

### 3.2. Optimization of Fermentation Parameters

The main goal for fermentation parameters optimization was to identify the optimal medium compositions and culture conditions, in relation to lipase production by *S. commune* UTARA1. Inoculums density (number of mycelia discs), moisture ratio (mL per g of SB), urea (%), incubation temperature (°C), SB solids (g), glucose (%), SB particle size (mm) and UCO ratio (mL per g of SB) used during solid state fermentation were the parameters under investigation. These factors were evaluated by the application of a 2-Level Fractional Factorial Design (Design-Expert^®^ 7.0.0, Stat-Ease, Minneapolis, MN, USA). Eight independent variables in sixteen combinations in triplicates were organized according to the 2-Level Fractional Factorial Design matrix (Table 5). For each variable, a high (+1) and a low (−1) level was tested.

The optimum condition of the significant screened factors was determined by 3-Level Factorial Design (Design-Expert^®^ 7.0.0). Two independent variables in nine combinations in triplicates were organized according to the 3-Level Factorial Design matrix (Table 6). From the results obtained, an equation of the optimum condition that supported the maximum production of lipase from SB impregnated with UCO by *S. commune* UTARA1 was generated and validated.

### 3.3. Crude Enzyme Extraction and Lipase Assay

Phosphate buffer (0.1 M, pH 7) was added to extract the crude enzyme at the buffer to SB ratio of 10:1. The flasks were agitated in orbital shaker at 200 rpm, 30 °C for 30 min before filtering through muslin cloth. The filtrate was then centrifuged at 13,000 rpm for 5 min. The supernatant was used as crude enzyme extract. The lipase was assayed and carried out using 2.5 mM 4-nitrophenyl laurate (NPL, Sigma) as substrate. The assay mixture was prepared with 0.2 mL of NPL dissolved in isopropanol, 1.6 mL of 0.1 M phosphate buffer (pH 7) and 0.2 mL of crude enzyme extract. The reaction was left to run at room temperature for 30 min. Prior to spectrophometric analysis, the tubes were centrifuged at 10,000 rpm for 10 min at 4 °C. The absorbance was read at 400 nm against an enzyme-free control. One unit of lipase activity is defined as the amount of enzyme which releases 1 μmol of *p*-nitrophenol per minute under the assay conditions. The extinction coefficient of NPL is ε = 8.4 mM^−1^·cm^−1^.

### 3.4. Partial Purification of Crude Enzyme Extract

The crude enzyme extract (600 mL) was subjected to stepwise ammonium sulfate precipitations from 30 to 90% saturations at 4 °C. The precipitates were harvested by centrifugation at 8000× *g* for 20 min. The pellet was dissolved in 5 mL of 0.1 mM phosphate buffer, pH 7.0 and dialyzed (10,000 MWCO SnakeSkin^®^ dialysis tubes, Thermo Scientific, Waltham, MA, USA) prior use. Each fraction was subjected to lipase and protein (Bradford reagent, Amresco, Solon, OH, USA) assays followed by SDS-PAGE.

### 3.5. Lipase Stability and Deactivation

The partially purified lipase was subjected to stability and deactivation tests. The lipase fractions were pre-incubated over a range of 20–70 °C and sampling at 30 min, 1, 2, 3, 4 and 5 h. The lipase fractions were then assayed as described in Section 3.3.

### 3.6. Crude Fish Oil Extraction

Fish viscera and unwanted parts (400 g) were obtained from the local market in Kampar (Perak, Malaysia). They were homogenized using a blender (Waring, Stamford, CT, USA). Equal parts of distilled water and hexane were added and the mixture was shaken at 200 rpm for 30 min at room temperature. Then, the slurry was left to settle down overnight at 4 °C before centrifugation at 8000× *g* for 20 min at 4 °C. The top hexane layer was recovered and it was centrifuged again at 8000× *g* for 20 min at 4 °C. Then the layer was subjected to rotary evaporator (Büchi, Flawil, Switzerland) for hexane removal and oil concentration. The recovered crude fish oil was stored in the dark at 4 °C.

### 3.7. Oil Hydrolysis and Glycerol Esterification

The partially purified lipase (3 mL) was subjected to hydrolysis using 300 µL of oil (crude fish oil, coconut oil) or 0.3 g of butter. The mixtures were incubated at 30 °C, shaking at 140 rpm for 24 h in a solvent free system. Samples were taken for analysis. Free glycerol is quantified after derivatization [24]. Potassium periodate solution, 10 mM (1.2 mL) was added to a sample (2 mL) and was shaken for 30 s. Then, 0.2 M acetylacetone solution (1.2 mL) was added. The mixture was incubated at 70 °C for 1 min, with manual stirring. Subsequently, the mixture was cooled under tap water and the absorbance was read at 410 nm against an enzyme-free control. Esterification was carried out using glycerol and fatty acids were mixed according to their molar ratios in a solvent free system. The partially purified lipase (4%) was added. The mixture was incubated at 30 °C, shaking at 140 rpm for 5 days. Samples were taken daily and evaluated using thin layer chromatography (TLC) analysis (TLC silica gel 60 F254 Al plates, Merck, Kenilworth, NJ, USA). The plates were then visualized under iodine vapor treatments and UV 254 nm.

## 4. Conclusions

Out of eight factors affecting the fermentation, only moisture and UCO ratios were found to be significant. The potential applications of *Sc*LipA and *Sc*LipB in their partial purified forms were presented in this study. The results obtained suggest that *Sc*LipA and *Sc*LipB have application potential in selective hydrolysis of lipids, TAG modifications, and biodiesel production and that they are *sn*-2 regioselective lipases. It is appealing to observe that under certain conditions, *Sc*LipA and *Sc*LipB offer a natural alternative for the production of value-added products, compared to the chemical hydrolysis or synthesis. The use of these partial purified lipases facilitates cost reduction and the reaction conditions are more environmental friendly. The synthesis and hydrolysis products of TAGs, MAGs and DAGs derivatives are commercially valuable products, especially in the cosmetics, drugs and food industries. These direct reactions without organic solvents can be an alternative for future industrial applications.

## Figures and Tables

**Table 1:** Statistical analysis of the model (ANOVA) for 2-Level Fractional Factorial Design.

Source	Sum of Squares	Degrees of Freedom	Mean Square	*F* Value	*p*-Value Prob. > *F*
Model	0.078	7	0.011	4.22	0.0014
A ^1^	9.663 × 10^−3^	1	9.663 × 10^−3^	3.64	0.0637
B ^2^	0.029	1	0.029	10.77	0.0021
C ^3^	4.760 × 10^−3^	1	4.760 × 10^−3^	1.80	0.1875
H ^4^	0.018	1	0.018	6.89	0.0122
AB	7.701 × 10^−3^	1	7.701 × 10^−3^	2.91	0.0959
AC	4.641 × 10^−3^	1	4.641 × 10^−3^	1.75	0.1930
AH	4.681 × 10^−3^	1	4.681 × 10^−3^	1.77	0.1912
Lack of fit	0.030	8	3.772 × 10^−3^	1.59	0.1658

^1^ Inoculum density; ^2^ moisture ratio; ^3^ urea; ^4^ UCO ratio.

**Table 2:** Statistical analysis of the model (ANOVA) for 3-Level Factorial Design.

Source	Sum of Squares	Degree of Freedom	Mean Square	*F*-Value	*p*-Value Prob. > *F*
Model	0.130	3	0.044	14.24	<0.0001
A ^1^	0.052	1	0.052	16.72	0.0005
B ^2^	0.059	1	0.059	19.20	0.0002
AB	0.013	1	0.013	4.27	0.0503
Lack of fit	0.027	5	5.404 × 10^−3^	2.22	0.0973

^1^ Moisture ratio; ^2^ UCO ratio.

**Table 3:** Purification table of crude enzyme extract using 30–90% (NH_4_)_2_SO_4_.

Step	Volume (mL)	Total Protein (mg)	Total Activity (U·L^−1^)	Specific Activity (U·mg^−1^)	Purification (Fold)	Yield (%)
Crude	600.0	159.18	14.22	0.089	1.00	100.00
30%	87.0	16.23	5.10	0.314	3.53	35.86
40%	28.9	3.76	0.88	0.234	2.63	6.19
50%	152.0	18.16	1.14	0.063	0.71	8.02
60%	108.0	11.21	0.81	0.072	0.81	5.70
70%	5.0	2.48	0.18	0.073	0.82	1.27
80%	7.5	9.20	0.17	0.018	0.20	1.20
90%	5.5	3.68	0.00	0.000	0.00	0.00
Leftover	500	73.88	0.00	0.000	0.00	0.00

**Table 4:** Glycerol production of oil hydrolysis by *Sc*LipA and *Sc*LipB per day.

Partial Purified Lipases	Substrates	Glycerol Production (μmol/mg_protein_/day)
*Sc*LipA	Crude fish oil	2.489 ± 0.03
	Coconut oil	0.405 ± 0.01
	Butter	0.236 ± 0.02
*Sc*LipB	Crude fish oil	0.460 ± 0.03
	Coconut oil	nd ^1^
	Butter	nd ^1^

^1^ Not detected.

**Table 5:** Experimental range and levels of independent variables in the 2-Level Fractional Factorial Design.

Variable	Level	
	−1	+1
Inoculum density (number of mycelia discs)	2	10
Moisture ratio (mL per g of SB)	1:1	1:4
Urea (%)	0.1	1.5
Temperature (°C)	28	35
SB solids (g)	2	6
Glucose (%)	0	1
SB particle size (mm)	0.5–0.85	1.5–2.0
UCO ratio (mL per g of SB)	1:0.1	1:4

**Table 6:** Experimental range and levels of independent variables in the 3-Level Factorial Design.

Variable	Level		
Moisture ratio (mL per g of SB)	1:1	1:2	1:4
UCO ratio (mL per g of SB)	1:0.1	1:1	1:4
